# Lateral Prefrontal Theta Oscillations Reflect Proactive Cognitive Control Impairment in Males With Attention Deficit Hyperactivity Disorder

**DOI:** 10.3389/fnsys.2020.00037

**Published:** 2020-06-18

**Authors:** Francisco Zamorano, Leonie Kausel, Carlos Albornoz, Claudio Lavin, Alejandra Figueroa-Vargas, Ximena Stecher, Diego Aragón-Caqueo, Ximena Carrasco, Francisco Aboitiz, Pablo Billeke

**Affiliations:** ^1^Laboratorio de Neurociencia Social y Neuromodulación, Centro de Investigación en Complejidad Social (neuroCICS), Universidad del Desarrollo, Santiago, Chile; ^2^Unidad de Imágenes Cuantitativas Avanzadas, Clínica Alemana de Santiago, Universidad del Desarrollo, Santiago, Chile; ^3^Departamento de Imágenes, Clínica Alemana, Facultad de Medicina, Universidad del Desarrollo, Santiago, Chile; ^4^Centro Interdisciplinario de Neurociencias, Pontificia Universidad Católica de Chile, Santiago, Chile; ^5^Facultad de Economía y Negocios, Universidad del Desarrollo, Santiago, Chile; ^6^Escuela de Medicina, Universidad de Valparaíso, Valparaíso, Chile; ^7^Laboratorio de Neurociencias Cognitivas, Departamento de Psiquiatría, Centro Interdisciplinario de Neurociencia, Pontificia Universidad Católica de Chile, Santiago, Chile

**Keywords:** contextual control, EEG, attentional deficit, dorsolateral prefrontal cortex, p300

## Abstract

Attention Deficit Hyperactivity Disorder (ADHD) is a common neuropsychiatric disorder in which children present prefrontal cortex (PFC) related functions deficit. Proactive cognitive control is a process that anticipates the requirement of cognitive control and crucially depends on the maturity of the PFC. Since this process is important to ADHD symptomatology, we here test the hypothesis that children with ADHD have proactive cognitive control impairments and that these impairments are reflected in the PFC oscillatory activity. We recorded EEG signals from 29 male children with ADHD and 25 typically developing (TD) male children while they performed a Go-Nogo task, where the likelihood of a Nogo stimulus increased while a sequence of consecutive Go stimuli elapsed. TD children showed proactive cognitive control by increasing their reaction time (RT) concerning the number of preceding Go stimuli, whereas children with ADHD did not. This adaptation was related to modulations in both P3a potential and lateral prefrontal theta oscillation for TD children. Children with ADHD as a group did not demonstrate either P3a or theta modulation. But, individual variation in theta activity was correlated with the ADHD symptomatology. The results depict a neurobiological mechanism of proactive cognitive control impairments in children with ADHD.

## Introduction

Attention Deficit Hyperactivity Disorder (ADHD) is the most widespread neuropsychiatric disorder in childhood, with an incidence of 10% to 12% (Faraone et al., 2006; Leung and Hon, 2016). Increasing evidence suggests that children with ADHD present a delay in their brain development (Shaw et al., 2007, 2012), and structural magnetic resonance imaging (MRI) studies have shown that the most affected area is the prefrontal cortex (PFC; Sonuga-Barke and Halperin, 2010; Shaw et al., 2012; Sripada et al., 2014). The degree of delay in maturation of the dorsolateral PFC and the medial PFC predicts the persistence of inattention symptoms during adulthood (Shaw et al., 2013). This delay in prefrontal maturation in children with ADHD is consistent with the current interpretation of ADHD symptoms, which states that the main features of the disorder are due to an impairment in behavioral and cognitive control mechanisms caused by deficient dopaminergic signaling (Swanson et al., 2007; Aboitiz et al., 2014). Thus, functional maturation of the PFC is reflected in the performance of a series of executive functions, including attentional control, working memory, inhibitory and cognitive control, which are usually impaired in ADHD subjects (Faraone et al., 2015). Interestingly, across the lifespan of patients with ADHD, these cognitive deficits are independent of one another, and it is currently unknown whether and how cognitive deficits drive ADHD symptoms (Sonuga-Barke et al., 2010). In this context, it is important to identify specific relationships between cognitive deficits, neurobiological mechanisms, and clinical symptomatology.

Proactive cognitive control is the ability to modify our responses anticipating the necessity of cognitive control based on the context provided by recent events (Hu and Li, 2012; Koechlin, 2014; Chang et al., 2017; Ryman et al., 2018), as opposed to reactive cognitive control, which requires the engagement of control processes at the onset of challenging task demands (Braver et al., 2009; Braver, 2012). Thus, proactive cognitive control requires the integration of past events and experience to adjust current goal-directed actions (Donoso et al., 2014), modifying and improving the cognitive control required for upcoming conflictive stimuli. An illustrative example is given by stop signal tasks such as the Go-Nogo task. In this task, the appearance of infrequent Nogo stimuli generates an inhibitory response that requires reactive cognitive control. Interestingly, this reaction can be improved by analyzing the context of the recent stimuli, because as more consecutive Go stimuli occur, there is a higher probability that a Nogo stimulus will occur in the next trial. This is the sequence effect. Thus, in this task, the proactive cognitive control is manifested by modifying the response (e.g., longer RTs) based on the context (e.g., the number of preceding Go stimuli; Zamorano et al., 2014).

The expectation of a target stimulus during an ongoing sequence of stimuli generates an adaptation of cognitive resources related to proactive cognitive control. Such a process is generated by the sustained activity of the lateral PFC (Ryman et al., 2018), interacting with medial prefrontal regions (Cavanagh and Frank, 2014). Studies using electroencephalography (EEG) have demonstrated that stimulus sequence context modulates brain activity, increasing the P3 event-related potential (ERP) component and low-frequency theta and delta oscillations in a linear progression as the number of preceding non-targets increase (Polich and Bondurant, 1997; Zamorano et al., 2014; Harper et al., 2016) Early functional MRI (fMRI) studies indicate that the progression of the sequence is related to an increase in activity in both lateral PFC and anterior cingulate cortex (ACC) in adults, while children do not present a clear sequence effect (Durston et al., 2002a,b, 2003; Kerns, 2006) although it must be kept in mind that the time resolution of the fMRI technique precludes a clear analysis of sequence effect (Zamorano et al., 2014).

Studies in typically developing (TD) children have found that an increase in the activity of the dorsal ACC (dACC) is related to performance monitoring in several tasks that require reactive cognitive control (Ordaz et al., 2013; Rubia, 2013; Luna et al., 2015). Following this, EEG studies have found that an increase in the error-related negativity with age (Segalowitz et al., 2010) is related to an increase in the amplitude of theta oscillation in the medial prefrontal cortex (Billeke et al., 2013b, 2014; Cavanagh and Frank, 2014). The medial prefrontal theta oscillation seems to be a cortical mechanism by which the need for cognitive control is monitored (Cavanagh and Frank, 2014). These medial structures are strongly coupled with the lateral prefrontal cortex, which subserves the implementation of cognitive control, for example, inhibitory control (Kerns, 2006; Shenhav et al., 2013). Interestingly, reactive and proactive cognitive control is also reflected in theta oscillation and BOLD activity in the lateral prefrontal cortex (Cavanagh et al., 2010; Braver, 2012; Billeke et al., 2013b; Larrain-Valenzuela et al., 2017). In contrast to medial prefrontal functioning, it is not clear how the lateral prefrontal activity matures during development. Prior works with fMRI in children have found both increases and decreases in the activity of the lateral prefrontal cortex related to cognitive control (Marsh et al., 2006; Ordaz et al., 2013; Rubia, 2013). Recent interpretations indicate that the maturation of these areas is related to more efficient cortical processing, which in turn is related to decreases in the BOLD signal (Luna et al., 2015). However, it is unknown how these observations are related to oscillatory activity in these regions in TD children and also in children with ADHD.

Here we tested the hypothesis that children with ADHD have impaired proactive cognitive control as compared to TD children and that this lack of contextual/proactive adaptation should be reflected in differences in the PFC oscillatory activity.

## Materials and Methods

### Participants

Fifty-four male children aged 8–13 (mean = 11.4 year, see Table 1), 29 children with ADHD, and 25 TD children, participated in our study. Since there is a higher prevalence in males than females, and sex-related differences in typical development and the proportion of clinical subtypes of ADHD, we recruited only males to reduce possible confounding sources of variability. All participants were right-handed, Chilean-Spanish speaking with normal or corrected-to-normal vision, and did not have color-vision deficiency. Children underwent a complete physical and psychological examination and were classified as children with ADHD or TD children using Conner’s Abbreviated Parent-Teacher Questionnaire and DSM-IV criteria for ADHD. All participants had an average or higher IQ (WISC-R) and did not have any major comorbidity (MINI-Kid; De la Peña et al., 2009; Sheehan et al., 2010).

**Table 1:** Demographics of the population.

	ADHD		TD		Diff
	mean	Std. dev	mean	Std. dev	*p*-value
*n*	29		25		
Age	11.3	1.7	11.66	1.4	0.5
IQ	103.7	7.9	108.30	12.8	0.06

Children with ADHD were recruited from general outpatient services. All of them had a clinically proven history of good response to stimulant medication (oral methylphenidate), which they had been using for at least 3 months when recruited. They were asked to interrupt stimulant treatment on the day of the experimental session. TD participants were selected out of a large group of children from public schools who volunteered for the study.

### Ethics Statement

Written consent was signed by parents and written assent was signed by the children after a detailed explanation of the scope of the study, following guidelines and procedures approved by the Ethics Committee of the Pontificia Universidad Católica de Chile. All experiments were performed at the Cognitive Neuroscience Laboratory of the University.

### Task

**Figure 1:**
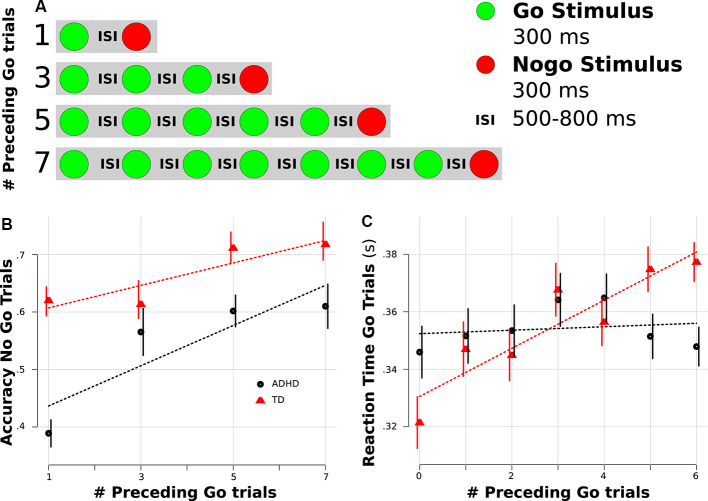
Behavioral results. **(A)** Task scheme. **(B)** Accuracy rate for Nogo trials separated by the number of preceding Go trials. **(C)** Reaction time (RT) for Go trials separated by the number of preceding Go trials. **(B,C)** Red color represents typically developing (TD) children, and black indicates Attention Deficit Hyperactivity Disorder (ADHD) children. Dotted lines represent linear regression fitting. Error bars represent standard error of the mean.

Participants performed the visual Go-Nogo task from Zamorano et al. (2014) while their brain activity was measured with electroencephalography (EEG). The experimental paradigm consisted of a serial presentation of screen centered green or red colored circles of 300 ms of duration (3.5 u of visual arc) that represented Go and Nogo stimuli respectively. Subjects were instructed to press a button as fast as possible after a Go stimulus (green circle) and not to press the button after a Nogo stimulus (red circle). Inter-stimulus intervals (ISI) were drawn from uniform distributions, generating 500–800 ms ISI (Figure 1A). Nogo stimuli were never presented consecutively. Nogo trials were preceded by sequences of 1, 3, 5, or 7 Go trials. This allowed us to evaluate proactive cognitive control, considering that when used, the context (number of preceding Go trials) should modify the response (RT on preceding Go trials). The task was programmed in Presentation 13.0 (Neurobehavioral Systems, Inc.) and the stimuli were presented on a 210 Cathode Ray Tube monitor positioned 57 cm in front of the subject. Each participant performed two blocks of 300 trials, which included ~75 Nogo trials (mean probability of Nogo trials was 0.25).

### Electrophysiological Recordings

Continuous EEG recordings were obtained with a 40-electrode NuAmps 10/20 EEG System (Neuroscan). All impedances were kept below 5 kΩ. Electrode impedance was re-tested during pauses to ensure stable values throughout the experiment. All electrodes were referenced to averaged mastoids during acquisition and the signal was digitized at 1 kHz. Electro-oculogram was obtained with four electrodes. All recordings were acquired using Scan 4.3 and stored for off-line analysis. At the end of each session, the electrode position was digitized using a 3D tracking system (Polhemus Isotrak). For all analyses, we used a 30 s-period acquired before the beginning of each block as the baseline.

### Behavioral Data Analysis

Accuracy and RT were analyzed with both mixed ANOVA and mixed linear regression. For ANOVA, we used condition (Go or Nogo stimuli), diagnosis (ADHD or TD), and the progression of Go trial sequences (1 or 3 or 5 or 7 go sequence) as factors. For mixed linear regression, we used condition (Go or Nogo stimuli) and diagnosis (ADHD or TD) as factors and the progression of Go trial sequences (1 or 3 or 5 or 7 go sequence) as a linear regressor. For mixed linear regression, we used single trials per subject and clustered the errors (variance) per subject including random effects for the intercept and regressors. Data were tested for normality with the Kolmogorov–Smirnov test. When the data did not meet the normal distribution assumption, nonparametric tests were used.

### EEG Data Analysis

EEG signals were processed using the LAN toolbox (Billeke et al., 2014). Data were preprocessed using a 0.1–100 Hz band-pass filter (4th order Butterworth, zero-phase shift filter). Eye blinks were identified by a threshold criterion of ± 100 μV, and their contribution was removed from each dataset using independent component analysis (using as criterion both the topography and single-trial time series of each component). Other remaining artifacts were detected by both automatic artifact detections (voltage threshold and amplitude threshold in the frequency domain) and visual inspection of the signal and the trials that contained them were removed. We obtained 53.5 Nogo and 209 Go artifact-free trial per subject [Nogo trial ADHD: 52.4 range (39–64), TD: 54.1 (40–64), *p* = 0.3; Go trial ADHD: 201.7 range (144–236), TD: 218.6 (161–239), *p* = 0.003].

Event-related potentials (ERP) were computed as the mean of the signal for each electrode and each participant over correct Nogo trials (the Nogo stimuli onset marked the time zero). P3a component was defined as the positive deflection with peak latency at about 380 ms post-stimulus onset and frontal topology. For the peak analyses, we explore for each subject the maximum positivity between 300–500 ms at the FCz electrode.

Induced power distribution was computed using Wavelet transform for correct trials. We applied a 5-cycle Morlet wavelet for a time window of −0.5 s to 1 s around the onset of each stimulus (range: 2–45 Hz in 1 Hz Step). Then, we used all of the artifact-free single trials for the modeling. For the time-frequency analyses, we used the dB of power related to a baseline during the fixation phase (10 s before the beginning of each block). We computed the models for each subject based on the single-trial wavelet transform (first-level analysis, see Billeke et al., 2015; Soto-Icaza et al., 2019).

$$\text{Power(f,t) = }{\text{b}}_{\text{1}}{\text{+b}}_{\text{2}}^{\text{*}}{\text{NoGo+b}}_{\text{3}}^{\text{*}}{\text{Sequence}}^{\text{*}}{\text{NoGo+b}}_{\text{4}}^{\text{*}}\text{Error(tr}-\text{1)}$$

where b_1_ is the intercept, and b_2_ is the slope or coefficient for the variable Nogo Stimuli (dummy variable that takes the value 1 when the presented stimulus was a Nogo, and 0 when the presented stimulus was a Go), b_3_ is the slope for the interaction between Nogo and Sequence (defined as the number of preceding Go stimuli in the sequence) and b_4_ is the slope for a dummy repressor indicating if an error occurs in the previous trials. The latter was used to control for reactive cognitive control. Thus, per each regressor and subject, we obtained a three-dimensional matrix (time, frequency, electrode), which we used in the second-level analysis. In this analysis, we compared each bin of the preceding matrices across subjects. We carried out two types of comparisons: (i) we explored for consistent modulations within the same condition and group. For this, we used the Wilcoxon signed-rank test, evaluating whether the means were other than zero (see upper and middle panels of Figure 3A); and (ii) we explored differences between groups. For this, we used the Wilcoxon sum rank test, evaluating whether the differences of the means between groups were other than zero for non–paired samples (see lower panel of Figure 3A). Then, for multiple comparison corrections, we used the Cluster-based Permutation (CBP) test.

**Figure 2:**
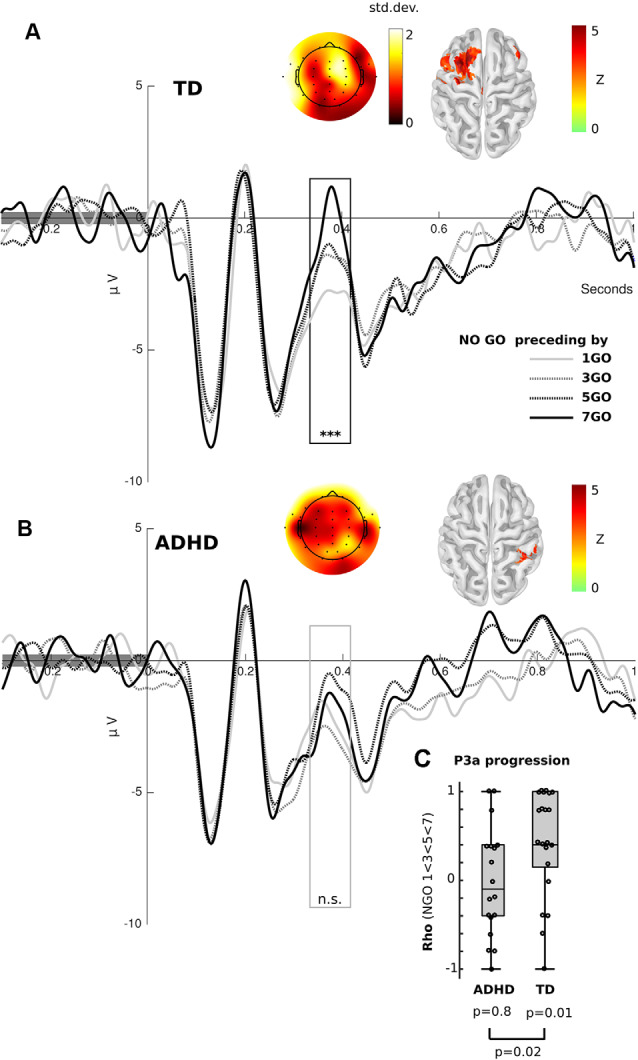
Event-related potential (ERP) results. **(A)** Frontal ERP elicited by Nogo stimuli separated by the number of preceding Go trials in TD children. The black box shows the significant differences between conditions [Kruskal–Wallis and Cluster-based Permutation (CBP) test, *p* < 0.01]. **(B)** Frontal ERP elicited by Nogo stimuli separated by the number of preceding Go trials in ADHD children. The gray box represents the P3a, where significant differences were found for the TD group, but not for the ADHD children (Kruskal–Wallis, *p* > 0.05, uncorrected). **(A,B)** Color in the scalp plot represents the standard deviation between conditions. In the cortex surface (source space), the *Z*-value of the difference among conditions is plotted. **(C)** Box plot of the spearman rho value of the correlation between P3a peak (FCz electrode) and the number of preceding Go stimuli per subject, separated by group. ***p < 0.001, n.s. = *p* > 0.05.

**Figure 3:**
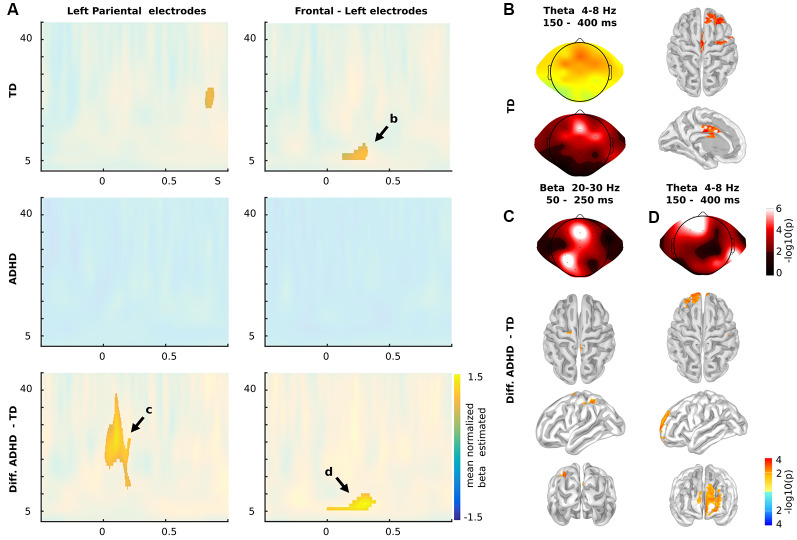
Time-frequency results. **(A)** Time-frequency charts for Nogo stimuli in Left Parietal (left panels) and frontal electrodes (right panels), for TD children (upper panels), children with ADHD (middles panels) and the differences between both groups (Diff: ADHD—TD; lower panels). **(B)** Theta oscillatory activity (4–8 Hz) between 150 and 400 ms post stimuli presentation in both scalp (mean of beta estimated and *p*-values) and source levels for TD children as indicated in **(A)**. **(C)** Statistical differences between TD and ADHD children in the Beta oscillatory activity as indicated in (**A**; 20–30 Hz, 50–250 ms). **(D)** Statistical differences between TD and ADHD children in the Theta oscillatory activity as indicated in **(A)**. **(A–C)** Color represents the mean across subjects of the normalize beta estimated (beat/standard error of the mean) of the individual correlation between the power of the oscillatory brain activity and the sequences of Go stadium that precede the Nogo stimulus. The clusters with significant effects are highlighted (*p* < 0.01 cluster-corrected). **(C,D)** Color represents the *p*-values of the differences between groups. **(B–D)** For source levels, color represents the *p*-values at a threshold of *q* < 0.05 (FDR correction).

### Permutation Test for Multiple Comparison Correction

To correct for multiple comparisons, we carried out the CBP test (Maris and Oostenveld, 2007). As we did not assume any *a priori* related to the topography of the modulation, all electrodes were used for the cluster detection. Clusters of significant areas were defined by pooling neighboring sites (bins of time-frequency-electrode matrices or sources) that showed the same effect (Cluster threshold detection; *p* < 0.05 for the statistical test carried out in each site of the time-frequency chart, e.g., Wilcoxon test). The cluster-level statistics were computed as the sum of the statistics of all sites within the corresponding cluster. We evaluated the cluster-level significance under the permutation distribution of the cluster that had the largest cluster-level statistics. The permutation distribution was obtained by randomly permuting the original data. For paired analysis, we permute the sequences of Go stimuli (1 or 3 or 5 or 7 preceding go stimuli). For unpaired analysis (group comparison) we permute the diagnosis factor (ADHD or TD group). After each permutation, the original statistical test was computed. After 1,000 permutations, the cluster-level significance of each observed cluster was estimated as the proportion of elements of the permutation distribution greater than the cluster-level statistics of the corresponding observed cluster.

### Source Estimations

Source estimations were carried out in BrainStorm (Tadel et al., 2011). We estimated the neural current density time series at each elementary brain location applying a weighted minimum norm to estimate inverse solution with unconstrained dipole orientations in single trials per condition per subject. We used a tessellated cortical mesh template surface derived from the default anatomy of the Montreal Neurological Institute (MNI/Colin27) warped to the individual head shape (using ~300 head points per subject) as a brain model to estimate the current source distribution. We defined 3 × 5,005 sources constrained to the segmented cortical surface (three orthogonal sources at each spatial location), and we computed both a three-layer (scalp, inner skull, outer skull) boundary element conductivity model and the physical forward model. The measured electrode level data *X*(*t*) = [*x*_1_(*t*),…, *x*_n_electrode_ (*t*)] is assumed to be linearly related to a set of cortical sources *Y*(*t*) = [*y*_1_ (*t*),…, *y*_m_source_ (*t*)] (3 × 5,005 sources, see above) and to an additive noise *N*(*t*): *X*(*t*) = *LY*(*t*) + *N*(*t*), where L is the physical forward model. The inverse solution was then derived as *Y*(*t*) = *WX* (*t*) = *RL*^T^ (*LRL*^T^ + *γ*^2^*C*)^−1^ *X*(*t*), where W is the inverse operator, R and C are the sources and noise covariance, respectively, and λ is the regularization parameter. R is the identity matrix that was modified to implement depth-weighing (weighing exponent 0.8). The regularization parameter λ was set to 1/3. To estimate cortical activity at the cortical sources, the recorded raw EEG time series at the sensors x(t) was multiplied by the inverse operator W to yield the estimated source current as a function of time at the cortical surface *Y*(*t*) = *WX*(*t*). Since this is a linear transformation, it does not modify the frequencies of the underlying sources. It is therefore possible to undertake time-frequency analysis on the source space directly. In this source space, we computed frequency decomposition using the Wavelets transform as in the scalp levels (see above). Since we used a small number of electrodes and no individual anatomy for head model calculation, the spatial precision of the source estimations was limited. To provide more information about the localization procedure, for all source estimations, we show the scalp distribution of the activity computed separately from the electrode space. Finally, to minimize the possibility of erroneous results, we only present source estimations when there are both statistically significant differences at the electrode level and when the differences at the source levels survive a multiple comparison correction [False discovery rate (FDR) *q* < 0.05].

## Results

### Behavioral Results

Both groups demonstrated similar RTs (ADHD: 353.2 ms; TD: 346.2 ms; Wilcoxon test *p* = 0.72) and accuracy (ADHD: 0.85, TD: 0.92, *p* = 0.29) in Go trials, whereas the ADHD group demonstrated a higher rate of commission errors during Nogo trials (accuracy ADHD: 0.55, TD: 0.66, *p* = 0.03). Since changes in RT and accuracy related to the position of the Go stimulus in a sequence could be an indicator of proactive cognitive control, we assessed for differences in this progression between groups. To this end, we used both a mixed linear regression (assuming a linear progression) and a mixed ANOVA (with no *a priori* assumption related to a specific linear progression). For RT during correct Go trials, we found a linear progression in the TD group, while no such progression was found in the ADHD group (see Figure 1C). This led to a significant modulation for the factor sequence (mixed ANOVA, *F* = 5, 68, *p* = 0.022; linear mixed model, *t*_(233)_ = 3.1, *p* = 0.001) and for the interaction between sequence and group (mixed ANOVA, *F* = 5, 3, *p* = 0.027; linear mixed model, *t*_(233)_ = −2.24, *p* = 0.026). To rule out possible confounding factors related to the slowing of reaction after an error (reactive cognitive control), we carried out an additional single-trial regression including if the preceding trial was an error or not (see “Materials and Methods” section). Interestingly in this regression, the interaction between sequence and group remains significant (*t*_(8253)_ = −2.2, *p* = 0.027). For the accuracy of Nogo trials, we found that both groups demonstrated a progression, but the ADHD group presented a lower rate (Figure 1B). Thus, the group factor was significant in both analyses (mixed ANOVA, *F* = 6.1, *p* = 0.018; linear mixed model, *t*_(233)_ = −2.27, *p* = 0.024), while the sequence factor was significant only in the ANOVA (mixed ANOVA, *F* = 7.11, *p* = 0.002; linear mixed model, *t*_(233)_ = 1.58, *p* = 0.11).

### Electrophysiological Results

Following the behavioral results, we assessed for electrophysiological modulations related to the progression of Go stimuli. To this end, we only used the progression of ERPs related to correct Nogo trials. This is because these trials generate a stronger electrophysiological response and do not include a motor-related activity. Additionally, it has been shown that in adults similar to progressions related to Go sequences occur in both Go and Nogo trials (Zamorano et al., 2014). Figure 2 shows the progression of ERP in both groups. Using the Friedman test and CBP test, we found that TD children presented an amplitude progression for the P3a component. As in adults, we found a significant cluster (*p* < 0.01) in frontal electrodes. In the source analysis, we found that this modulation was bilateral, although with a predominance of left hemisphere activity (FDR *q* < 0.05). Interestingly, we did not find any significant cluster in the ADHD group. Using a permissive threshold (uncorrected *p* < 0.05) for the source analysis, we found a modulation in the right sensory-motor cortex in the same time-windows where controls presented significant P3a modulations. Comparing the progression in both groups, we extracted amplitude values at the peak of the P3a component (FCz electrode) and then carried out a correlational analysis per subject testing a monotonic increase (Spearman correlation, P3a elicited by Nogo preceding by 1 Go < 3 Go < 5 Go < 7 Go stimuli). At the group level, TD children presented rho values that were greater than zero (Wilcoxon test, *p* = 0.01) and greater than those of ADHD children (*p* = 0.02, see Figure 2C). This indicates that TD children, as opposed to children with ADHD, had significantly increased P3a amplitude with longer sequences of preceding Go trials.

Since recent reports have shown modulations in a lower oscillatory activity such as delta and theta during the processing of contextual sequence information and proactive cognitive control during inhibition (Harper et al., 2016; Ryman et al., 2018), we tested for time-frequency modulations in our subjects. We calculated the single-trial wavelet transform (−0.5 to 1-s post-stimulus onset) for each subject and modeled the power of the signal using Nogo stimuli, number of preceding Go stimuli and if the preceding trial was an error as regressors (see “Materials and Methods” section for more details). As in the ERP analysis, we studied the oscillatory modulation related to the sequence of Go trials that preceded the current Nogo trial (Figure 3). We found that TD children presented a significant modulation in the theta band (4–8 Hz) that occurred between 150 and 400 ms post-stimulus onset in left frontal electrodes.

**Figure 4:**
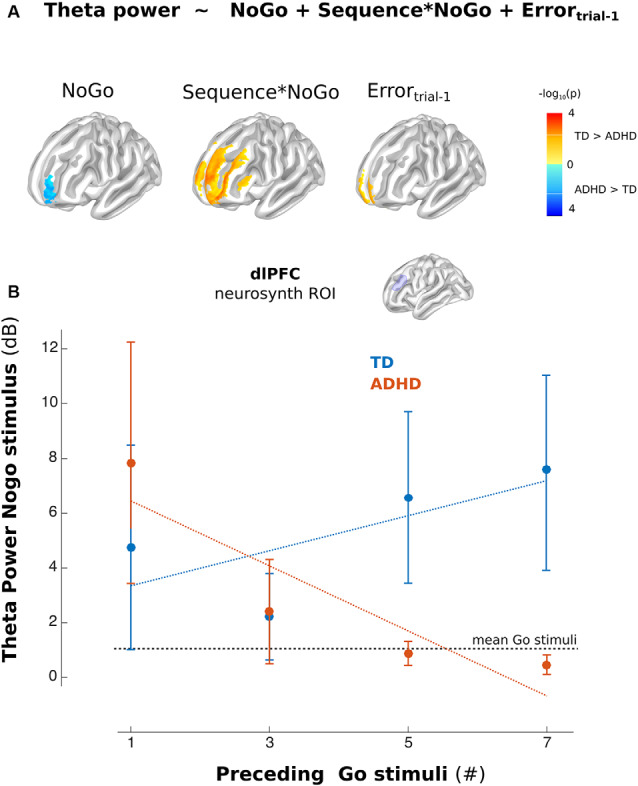
Theta activity in the left Lateral PFC (lPFC). **(A)** Model used in Time-frequency analysis. The color indicates the differences between the corresponding regressors between groups (Blue ADHD>TD and Red TD>ADHD, *p* < 0.01 uncorrected). **(B)** Mean of the power of the lPFC (dorsolateral PFC from Neurosynth) ROI for Nogo stimuli related to Go stimuli separated per sequence (illustrative propose). Error bars represent standard error of the mean.

The source analysis showed this modulation took place over the middle and superior frontal gyrus (Lateral PFC, lPFC), precentral gyrus, and midcingulate cortex. At the scalp level, the ADHD group did not demonstrate significant modulations. This fact led to significant differences between groups. These differences mainly took place in the theta modulation over lPFC (Figure 4A). However, when including Nogo stimulus into the regression model, the model showed more theta activity in children with ADHD than in TD children. Figure 4B shows the power of theta activity in lPFC (dorsolateral PFC ROI selected from the left peak of the online meta-analysis using neurosynth^1^) for each Nogo trial/Go sequences combination per group. Thus, ADHD children failed to adjust their oscillatory activity to the context of the sequence of preceding Go stimuli (Figure 4). Interestingly, the mean *T*-value of the contextual modulations of the left lPFC ROI, correlated with the Conner’s scale of the ADHD symptomatology (rho = −0.72, *p* = 8e-6).

## Discussion

The diagnosis of ADHD has been associated with a broad plateau of symptoms and cognitive deficits that goes from impairments in working memory to motor control (Nikolas and Nigg, 2013). Recent research has emphasized poor behavioral inhibition as a central factor behind the different expressions of the syndrome (Aboitiz et al., 2014). In this work, we used the Go-Nogo task to observe how participants integrated context information to accurately respond to the Go stimuli and inhibit their response to the Nogo trials. The behavioral results showed that children with ADHD do not present longer RTs on Go trials related to the amount of preceding Go trials. This means that when the context indicated that there was an increase in the probability of Nogo stimuli, ADHD children did not adjust their cognitive control accordingly. This lack of RT progression suggests that children with ADHD, unlike TD children, do not develop an expectation of the target stimulus (the Nogo stimulus). This could be related to impairments in the allocation of cognitive resources for proactive cognitive control (Braver, 2012; Chang et al., 2017). This lack of contextual control for inhibiting the motor response in the Nogo trials contributes to the higher error rate of the ADHD group in the Nogo trials, a finding that is consistent with previous evidence (Spronk et al., 2008; Henríquez-Henríquez et al., 2015). This deficit in inhibitory functioning has been related to delayed maturational processing, consistent with studies in healthy children that show that younger children have a poorer performance than older ones on this type of task (Yong-Liang et al., 2000; Durston et al., 2003; Spronk et al., 2008). Interestingly, recent reports have indicated that impaired proactive cognitive control is also present in other pathologies such as schizophrenia and depression in which these alterations crucially impact the quality of life of these patients (Billeke and Aboitiz, 2013; Billeke et al., 2013a; Solomon et al., 2014; Ryman et al., 2018).

Consistent with the behavioral results, TD children showed an increased progression of P3a amplitude during successive Nogo stimuli related to the number of preceding Go trials. The modulation of this component, traditionally associated with the allocation of attentional resources (Polich and Bondurant, 1997) suggests that TD children adapt their expectations of the appearance of a Nogo trial given the repetition of Go trials. The absence of P3 modulation in ADHD is in line with evidence showing reduced P3 amplitude in children with ADHD performing attention-related tasks (Spronk et al., 2008), which has been interpreted as signs of delayed attention and response inhibition development (Bruin and Wijers, 2002). Other studies have also shown a decrease in the amplitude of Nogo-P3 in young healthy children when compared to older children. This decrease is associated with poorer performances and immature response inhibition processing (Jonkman, 2006). Moreover, the Nogo-P3 amplitude can be enhanced by priming the responses, which increases the expectation (Bruin et al., 2001; Bruin and Wijers, 2002). In this sense, the reduced P3 progression suggests that children with ADHD do not integrate the number of Go trial repetition as task-relevant information such as TD children do.

A broader interpretation of the modulation of the P3 component in sequential stimuli presentation is taking this response as a mixture of separable theta and delta activity, which is related to time-frequency activity in Go-Nogo paradigms (Polich, 2007; Bernat et al., 2015). Our results show oscillatory modulations related to proactive cognitive control in TD, but not in children with ADHD. A similar effect has been found previously in TD children in delta frequency also related to the Nogo P3 component (Polich, 2007; Bernat et al., 2015). Interestingly, children with ADHD did show theta power as TD children, but they failed to adjust this activity to the sequential context of Go stimuli. This can be interpreted as a reactive strategy rather than a proactive one (Braver et al., 2009). The source analysis revealed that the difference in the theta oscillations related to the stimuli sequence in TD children took place in the lPFC, a region traditionally associated with inhibition signaling (Mueller et al., 2017; Zamorano et al., 2017). There is evidence that suggests that children with ADHD have impaired contextual signaling in cognitive control for inhibitory responses. One example is that children with ADHD have poorer performance on social and economic decision-making tasks, showing the absence of error-related response modulations that are associated with contextual information (Billeke et al., 2014, 2020; Gonzalez-Gadea et al., 2016). Thus, the absence of P3 and oscillatory responses of ADHD children due to the sequential stimuli presentation might suggest that they do not integrate this information (the sequence of Go trials) as relevant for the preparation of the inhibitory response to the Nogo stimuli. This is in line with evidence suggesting that the causes of the behavioral inhibition impairment in children with ADHD are not necessarily related to a broad inhibition deficit but to the regulation of such responses related to the integration of critical information (Yong-Liang et al., 2000). In this context, it is relevant to mention reported working memory deficits in children with ADHD (Nikolas and Nigg, 2013). Working memory crucially depends on theta oscillation (Billeke et al., 2017; Larrain-Valenzuela et al., 2017; Figueroa-Vargas et al., 2020), and its impairment can preclude the adequate information maintaining necessary to exert general proactive cognitive control. In a broad sense, these functional results can be related to the functional differences observed in children with ADHD in prefrontal areas when compared to TD children (Nakao et al., 2011; Frodl and Skokauskas, 2012) and the difference between TD children according to age (Bruin et al., 2001; Jonkman, 2006; Spronk et al., 2008). Thus, the evidence presented here provides an important link between the previously reported differential brain development in children with ADHD, and their functional correlates that are in line with the clinical symptomatology. Interestingly, proactive cognitive control can be improved by training (Braver et al., 2009). This means that our results could be used to design training interventions and/or non-invasive brain stimulations that could help to improve this process in children with ADHD.

It is important to note some limitations of the current study. Source localization using EEG signals has several issues that decrease spatial precision. Future studies using magnetoencephalography or concomitant fMRI/EEG to precise the sources of the oscillatory mechanisms described here in more detail are needed. Additionally, this research was carried out only in male subjects. It is important to replicate these results in a sample of both females and males.

In conclusion, the current study is the first examination of the neurobiological mechanisms of proactive cognitive control processes in children with ADHD. Results indicate that children with ADHD fail to increase P3a and theta oscillation related to the context, which in TD children is related to slower RTs when the expectation of a target stimulus increases. Altered theta oscillation in children with ADHD was related to their symptomatology, suggesting that this failure is linked to relevant behavioral impairments in real-world settings. The conclusions obtained by experimental studies are usually constrained by the tasks and measures used to conduct the studies, which makes it difficult to integrate the findings in clinical practice. However, disentangling the precise neurobiological and cognitive mechanisms underlying the symptomatology can give crucial information for adequate therapeutic interventions that can improve the quality of life of these patients. Thus, the consistent behavioral and neural findings related to the dysfunction in context-dependent cognitive control of children with ADHD might help to provide a useful framework for entangled observed symptomatology to neurobiological models to enrich the diagnosis, and more importantly, to improve the treatments of this developmental disorder.

## Data Availability Statement

The datasets generated for this study are available on request to the corresponding author.

## Ethics Statement

The studies involving human participants were reviewed and approved by Comité de Ética de la Escuela de Medicina de la Pontificia Universidad Católica de Chile. Written informed consent to participate in this study was provided by the participants’ legal guardian/next of kin.

## Author Contributions

FZ, PB, and FA: experimental design. FZ, PB, and XC: carry out experiments. FZ, PB, LK, DA-C, and XS: data analysis. FZ, PB, CL, CA, AF-V, and FA: wrote article.

## Conflict of Interest

The authors declare that the research was conducted in the absence of any commercial or financial relationships that could be construed as a potential conflict of interest.

## Footnotes

^1^https://neurosynth.org
